# Association between weight-adjusted waist index and non-alcoholic fatty liver disease: a population-based study

**DOI:** 10.1186/s12902-024-01554-z

**Published:** 2024-02-18

**Authors:** Changhui Yu, Shiming He, Maobin Kuang, Chao Wang, Xin Huang, Guotai Sheng, Yang Zou

**Affiliations:** 1 Jiangxi Medical College, Nanchang University, Jiangxi Provincial People’s Hospital, Nanchang, 330006 Jiangxi China; 2 Jiangxi Cardiovascular Research Institute, Jiangxi Provincial People’s Hospital, The First Affiliated Hospital of Nanchang Medical College, Nanchang, 330006 Jiangxi China; 3 Jiangxi Provincial Geriatric Hospital, Jiangxi Provincial People’s Hospital, The First Affiliated Hospital of Nanchang Medical College, Nanchang, 330006 Jiangxi China

**Keywords:** Weight-adjusted waist index, Non-alcoholic fatty liver disease, NAFLD, Association, WWI

## Abstract

**Background:**

Obesity is the most important driver of non-alcoholic fatty liver disease (NAFLD); nevertheless, the relationship of weight-adjusted waist index (WWI), a new obesity index, with NAFLD is unclear.

**Methods:**

This retrospective study used data from the NAGALA project from 1994 to 2016. WWI values were calculated using waist circumference (WC) and weight measurements of the participants. Three stepwise adjusted logistic regression models were developed to assess the relationship of WWI with NAFLD in the whole population and in both sexes. Additionally, we also conducted a series of exploratory analysis to test the potential impact of body mass index (BMI), age, smoking status and exercise habits on the association of WWI with NAFLD. Receiver operating characteristic (ROC) curves were used to estimate cut-off points for identifying NAFLD in the entire population and in both sexes.

**Results:**

The current study included a population of 11,805 individuals who participated in health screenings, including 6,451 men and 5,354 women. After adjusting for all non-collinear variables in the multivariable logistic regression model, we found a significant positive correlation of WWI with NAFLD. For each unit increase in WWI, the risk of NAFLD increased by 72% in the entire population, by 84% in men, and by 63% in women. Furthermore, subgroup analyses revealed no significant discrepancies in the correlation of WWI with NAFLD across individuals with varying ages, exercise habits, and smoking status (all *P*-interaction > 0.05), except for different BMI groups (*P*-interaction < 0.05). Specifically, compared to the overweight/obese group, the relationship of WWI with NAFLD was significantly stronger in the non-obese group, especially in non-obese men. Finally, based on the results of ROC analysis, we determined that the WWI cut-off point used to identify NAFLD was 9.7675 in men and 9.9987 in women.

**Conclusions:**

This study is the first to establish a positive correlation between WWI and NAFLD. Moreover, assessing the influence of WWI on NAFLD in individuals without obesity may yield more valuable insights compared to those who are overweight or obese.

**Supplementary Information:**

The online version contains supplementary material available at 10.1186/s12902-024-01554-z.

## Background

NAFLD, the prevailing chronic liver disease globally, encompasses a range of liver conditions varying from uncomplicated steatosis to fibrosis [1–3]. As the metabolic syndrome manifestation in the liver, NAFLD is not only linked to the progression of liver cirrhosis and hepatocellular carcinoma but is also closely linked to the occurrence of cardiovascular diseases, chronic kidney disease, diabetes and malignancies [1, 4–8]. Alarming trends emerge as lifestyle shifts and obesity rates soar, causing a drastic surge in NAFLD prevalence. Relevant data showed an escalation from 25.3% (1990–2006) to 38.2% (2016–2019), impacting roughly one-third of the world's population [2]. This presents a significant challenge to healthcare systems [3, 9]. Therefore, NAFLD risk screening in the general population is very necessary.

As we all know, obesity is an important risk factor for NAFLD [3, 10, 11], and especially the existence of central obesity is closely related to the occurrence of NAFLD [1, 12]. Previously, various obesity-related indices have been widely used to identify or predict NAFLD. Many obesity indices, including the commonly used waist circumference (WC) and BMI, have been shown to be independently associated with NAFLD [13, 14]. However, it is important to note that BMI cannot differentiate fat distribution [15], and there are limitations in using BMI due to the influence of the "obesity paradox" [16–19]. Additionally, WC, although highly correlated with BMI, may not be suitable as a substitute for BMI [20].

WWI is a recently proposed new anthropometric index, which exhibits a weaker correlation with BMI and reduces the risk of the "obesity paradox" associated with BMI [21]. Furthermore, high WWI not only reflects both low muscle mass and high-fat mass in the body simultaneously [22], but also can be used to evaluate the subcutaneous and visceral fat area [23]. Previous studies have found strong associations of WWI with cardiovascular diseases, type 2 diabetes, hyperuricemia, and adult urinary protein excretion [21, 24–29]. However, the research on NAFLD and WWI is limited, with only one study published in May 2023 reporting an independent association between hepatic steatosis and WWI in a population of Americans [30]. Therefore, this study aimed to further explore the association of WWI with NAFLD using data from the NAGALA project.

## Methods

### Data source and study design

The data for current research was obtained from the NAGALA project. In summary, the NAGALA research project began in 1994 and recruited and collected data from the general population participating in the Human Dockyard Examination Program at the Murakami Memorial Hospital in Japan. The project's objective was to identify and evaluate long-term health conditions and associated risk elements. The study design has been described in more detail in other publications [31]. Additionally, the research data from the study has been made publicly available on the DRYAD public database by Okamura and colleagues [32]. In accordance with the Dryad Terms of Service, the data can be used for secondary analysis with new research hypotheses. It is important to note that the implementation of the NAGALA project was authorized by the Murakami Memorial Hospital Ethics Committee, and informed consent for data usage was obtained from each participant [31]. The current study is a post-hoc analysis based on the data from the NAGALA project, and the research protocol and design were authorized by Jiangxi Provincial People's Hospital Ethics Committee (IRB 2021–066).

**Fig. 1:**
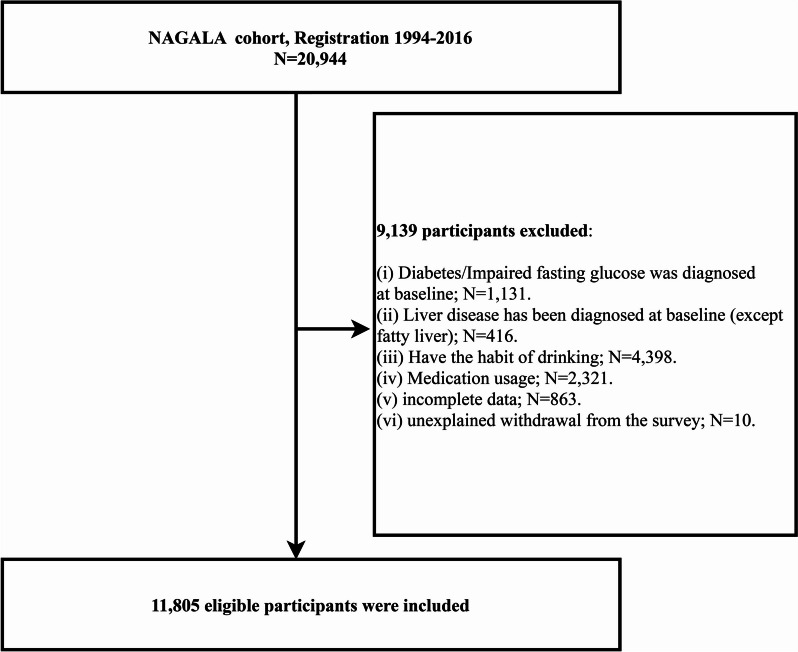
Flow chart for inclusion and exclusion of study participants

This study’s purpose was to assess the relationship of NAFLD with WWI using the NAGALA dataset. With the new research hypothesis, we conducted a cross-sectional design and included 20,944 individuals who underwent medical examinations between 1994 and 2016. Among these individuals, we further excluded participants with a diagnosis of diabetes or liver disease (excepted fatty liver) at baseline, as well as those with FPG > 6.1 mmol/L, using medications, having the habit of drinking, and covariable data missing, resulting in a final analysis cohort of 11,805 participants (Fig. 1).

### Data collection and measurements

Experienced healthcare professionals measured and recorded various anthropometric measurements of the participants, including WC, height, weight, and arterial blood pressure. BMI was calculated on the basis of weight and height. WWI was calculated as $\frac{{\text{WC}}({\text{cm}})}{\sqrt{Weight(kg)}}$ [21].

Information on age, drinking status, sex, smoking status, and exercise habits was collected through a questionnaire. The classification of smoking status included individuals who were not smokers, those who used to be smokers, and individuals who were currently smokers. Exercise habits were determined based on engaging in any type of physical activity at least once per week.

After fasting for 8 h, venous blood samples were collected from the antecubital vein of the subjects and stored in siliconized glass tubes containing sodium fluoride. These samples were then centrifuged immediately and stored at -80 degrees Celsius until analysis. Blood tests were carried out using a modular analysis system (Hitachi High-Technologies Corp., Ltd., Tokyo, Japan), which involved measuring biochemical parameters such as high-density lipoprotein cholesterol (HDL-C), aspartate aminotransferase (AST), fasting plasma glucose (FPG), gamma-glutamyl transferase (GGT), total cholesterol (TC), alanine aminotransferase (ALT), and triglycerides (TG). According to the manufacturer's data, the coefficients of variation of the above biochemical parameters are 1.7%, 1.9%, 2.3%, 2.5%, 2.1%, 2%, 2.3% and 1.2%, respectively. Additionally, glycated hemoglobin A1c (HbA1c) was measured using high-performance liquid chromatography.

### Evaluate NAFLD

The professional ultrasound technician examined the participants' abdomen by color ultrasound. To avoid selection bias, gastroenterologists did not have access to participants' clinical information when making the diagnosis. They evaluated several key items, including liver brightness, liver-kidney echo contrast, deep attenuation and vascular blurring based on abdominal ultrasonography, and made a diagnosis of NAFLD [33].

### Statistical analysis

First, subjects were divided into 2 groups according to gender, and the distribution type of continuous variables was assessed using QQ plots. Categorical variables were shown in the baseline table as frequency (percentage), and continuous variables were shown as median (interquartile distance) or mean (standard deviation); Chi-square test, Mann–Whitney U test, and t-test were used for between-group comparisons.

Second, we used multivariate linear regression to assess the collinearity between WWI and covariates (Supplementary Table 1) [34], and then performed multivariate logistic regression models with stepwise adjustment of non-collinear variables to determine the relationship of NAFLD with WWI. The results were presented as ORs with 95% confidence intervals (CIs). Model 1 firstly made preliminary adjustments for sex, BMI and age. Model 2 further adjusted for participants' lifestyle habits (drinking status, smoking status, exercise habits). Model 3 included adjustments for all non-collinear variables. Subsequently, based on Model 3, we conducted four sensitivity analyses: Sensitivity-1 excluded participants with exercise habits at baseline, Sensitivity-2 excluded participants over 60 years old at baseline, Sensitivity-3 excluded participants with systolic/diastolic blood pressure ≥ 140/90 mmHg at baseline, and Sensitivity-4 only participants with baseline BMI less than 25 kg/m^2^ were included. Sensitivity-5 excluded patients with hypertriglyceridemia at baseline. After determining the association between WWI and NAFLD, we further used restricted cubic spline (RCS) to fit and visualize the association between WWI and NAFLD. It is noteworthy that, considering the significant differences between sexes in terms of body composition, shape, and energy metabolism [35], the aforementioned correlational analyses were conducted separately within the entire population and within each gender.

Third, we performed exploratory stratified logistic regression analysis to test the relationship of WWI with NAFLD in different ages, BMI, smoking status, and exercise habits subgroups and examined differences among subgroups using likelihood ratio tests. In addition, we also used the ROC curve to analyze the recognition value of WWI to NAFLD in the whole population, men and women, and calculated the corresponding area under the curve, cut-off point, sensitivity and specificity. Furthermore, we applied ROC curves to analyze the discriminative value of WWI for NAFLD in the entire cohort, as well as in males and females separately. This included calculating the area under the curve (AUC), cut-off points, sensitivity, and specificity for each respective group.

In this research, all data analyses were conducted using R software (version 3.4.3) and Empower Stats (version 2.0), and the significance standard was set to *P* < 0.05 (two-sided).

## Results

### Baseline information

Eleven thousand eight hundred five participants were eventually included in the current study, including 6,451 men and 5,354 women. Table 1 shows the baseline characteristics of the study population grouped by gender. It can be seen that at baseline, male participants had significantly higher levels of weight, height, BMI, WC, ALT, AST, GGT, TC, TG, FPG, SBP, DBP and lower levels of WWI and HDL-C than those of the female population. In addition, male participants had significantly more smoking habits and a significantly higher prevalence of NAFLD (30.31% vs 7.21%).

**Table 1:** Baseline characteristics of the study population are summarized according to sex group

	**Women**	**Men**	***P*-value**
**No of subjects**	6451	5354	
**Age, years**	42.00 (37.00–49.00)	41.00 (36.00–49.00)	0.843
**Weight, kg**	51.60 (47.20–56.70)	66.40 (60.60–73.10)	< 0.001
**Height, m**	1.58 (0.05)	1.71 (0.06)	< 0.001
**BMI, kg/m**^**2**^	21.03 (2.95)	23.10 (3.09)	< 0.001
**WC, cm**	71.66 (8.11)	80.32 (8.10)	< 0.001
**WWI**	9.89 (0.68)	9.80 (0.51)	< 0.001
**ALT, IU/L**	14.00 (11.00–17.00)	20.00 (15.25–29.00)	< 0.001
**AST, IU/L**	16.00 (13.00–19.00)	18.00 (15.00–23.00)	< 0.001
**GGT, IU/L**	12.00 (10.00–14.00)	18.00 (14.00–25.00)	< 0.001
**HDL-C, mmol/L**	1.60 (1.37–1.86)	1.22 (1.03–1.45)	< 0.001
**TC, mmol/L**	5.09 (0.88)	5.16 (0.86)	< 0.001
**TG, mmol/L**	0.56 (0.40–0.81)	0.91 (0.63–1.37)	< 0.001
**HbA1c, %**	5.19 (0.32)	5.19 (0.32)	0.639
**FPG, mmol/L**	5.15 (0.41)	5.20 (0.41)	< 0.001
**SBP, mmHg**	114.28 (14.87)	115.40 (14.74)	< 0.001
**DBP, mmHg**	71.43 (10.36)	72.28 (10.27)	< 0.001
**Exercise habits**	1018 (15.78%)	955 (17.84%)	0.003
**Smoking status**			< 0.001
**non**	5750 (89.13%)	2157 (40.29%)	
**Former**	354 (5.49%)	1458 (27.23%)	
**Current**	347 (5.38%)	1739 (32.48%)	
**NAFLD**	465 (7.21%)	1623 (30.31%)	< 0.001

Values were expressed as mean (SD) or medians (quartile interval) or n (%)

*Abbreviations*: *NAFLD* non-alcoholic fatty liver disease, *BMI* body mass index, *WC* waist circumference, *ALT* alanine aminotransferase, *AST* aspartate aminotransferase, *GGT* gamma-glutamyl transferase, *HDL-C* high-density lipoprotein cholesterol, *TC* total cholesterol, *TG* triglyceride, *HbA1c* hemoglobin A1c, *FPG* fasting plasma glucose, *SBP* systolic blood pressure, *DBP* diastolic blood pressure, *WWI* weight-adjusted-waist index

### Association of WWI with NAFLD and sensitivity analysis

Significant positive correlations of NAFLD with WWI were observed in multivariate logistic regression models in all the whole population and men or women (Models 1–3) (Table 2). Although the associations weakened to some extent with stepwise adjustment of covariates, the positive correlation between WWI and NAFLD remained unchanged. After adjusting for all non-collinear variables, for every additional unit of WWI, the risk of NAFLD increased by 72% in the whole population, 84% in men and 63% in women.

**Table 2:** Logistic regression analyses for the association between WWI and NAFLD

	OR (95% CI)			
	Crude model	Model 1	Model 2	Model 3
WWI (All population)	2.78 (2.56, 3.01)	2.21 (1.94, 2.51)	2.19 (1.92, 2.50)	1.72 (1.49, 1.98)
Sex				
Men	5.04 (4.40, 5.78)	2.65 (2.21, 3.18)	2.60 (2.17, 3.13)	1.84 (1.50, 2.26)
**Women**	4.09 (3.54, 4.73)	1.78 (1.48, 2.14)	1.78 (1.49, 2.15)	1.63 (1.34, 1.98)

Model 1 adjusted for sex, age, height and BMI

Model 2 adjusted for sex, age, height, BMI, smoking status and exercise habits

Model 3 adjusted for sex, age, height, BMI, smoking status, exercise habits, ALT, AST, GGT, HDL-C, TC, TG, FPG, HbA1c and DBP

Note: Sex itself is not adjusted in the analysis based on sex stratification

*Abbreviations*: *WWI* weight-adjusted-waist index, *CI* confidence interval, *OR* Odds ratios

Furthermore, based on Model 3, we carried out five sensitivity analyses. The sensitivity analysis results were consistent with the main results, indicating a significant positive correlation between WWI and NAFLD, with increasing NAFLD risk as WWI increased (Table 3). These findings further confirmed the relatively stable positive correlation of WWI with NAFLD.

**Table 3:** Adjusted odds ratios and 95% confidence intervals for NAFLD risk associated with the WWI in different test populations: sensitivity analysis

	**OR** (95%CI)		
	**A**ll population	Men	Women
Sensitivity-1	1.73 (1.48, 2.01)	1.95 (1.56, 2.44)	1.57 (1.27, 1.95)
Sensitivity-2	1.95 (1.69, 2.24)	2.07 (1.69, 2.53)	1.85 (1.51, 2.27)
Sensitivity-3	1.73 (1.50, 2.00)	1.88 (1.53, 2.32)	1.61 (1.32, 1.97)
Sensitivity-4	2.70 (2.29, 3.19)	3.27 (2.59, 4.14)	2.16 (1.70, 2.73)
Sensitivity-5	2.91 (2.54, 3.33)	3.34 (2.72, 4.09)	2.60 (2.16, 3.12)

Adjusted for sex, age, height, BMI, drinking status, smoking status, exercise habits, ALT, AST, GGT, HDL-C, TC, TG, FPG, HbA1c and DBP

Note 1: (1) sensitivity-1: excluding subjects with exercise habits at baseline; (2) sensitivity-2: excluding subjects more than 60 years of age at baseline; (3) sensitivity-3: excluding subjects whose baseline SBP ≥ 140 mmHg or DBP ≥ 90 mmHg; (4) sensitivity-4: excluding subjects whose baseline BMI ≥ 25 kg/m^2^; (5) sensitivity-5: excluding subjects whose baseline TG ≥ 1.7 mmol/L

Note 2: Habit of exercise was not included in sensitivity-1; Age was not included in sensitivity-2; BMI was not included in sensitivity-4; TG was not included in model 4 of sensitivity-5

*Abbreviations*: *WWI* weight-adjusted-waist index, *CI* confidence interval, *OR* Odds ratios

### Subgroup analysis

After confirming the relationship of WWI with NAFLD, we further explored the differences in this association among different subgroups; notably, stratified analysis was separately carried out in the whole population and in men and women. According to the common clinical cut-off points, we stratified BMI and age, while the stratification methods mentioned earlier were used for smoking status and exercise habits. The new analysis results (Table 4) found no significant differences in the relationship of WWI with NAFLD among different subgroups based on age, habits of exercise, and smoking status, except for different BMI groups (P-interaction < 0.05). Specifically, compared to the overweight/obese group (BMI ≥ 25 kg/m^2^), the correlation of NAFLD with WWI was higher in the non-obese group (BMI < 25 kg/m^2^).

**Table 4:** Stratified associations between WWI and NAFLD by age, sex, BMI, exercise habits, drinking status and smoking status

	**All population**		**Men**		**Women**	
Subgroup	adjusted OR (95%CI)	*P-*interaction	adjusted OR (95%CI)	*P-*interaction	adjusted OR (95%CI)	*P-*interaction
Age (years)		0.1455		0.7311		0.1845
18–44	1.84 (1.47, 2.29)		2.07 (1.56, 2.73)		1.61 (1.11, 2.33)	
45–59	1.85 (1.53, 2.24)		1.89 (1.39, 2.57)		1.77 (1.39, 2.27)	
≥ 60	1.01 (0.57, 1.79)		1.43 (0.57, 3.61)		0.83 (0.38, 1.78)	
BMI (kg/m^2^)		0.0016		< 0.0001		0.0290
< 25	2.59 (2.23, 3.02)		3.21 (2.56, 4.01)		2.38 (1.89, 3.00)	
≥ 25	1.78 (1.45, 2.19)		1.53 (1.13, 2.09)		1.56 (1.15, 2.12)	
Exercise habits		0.8434		0.2454		0.5225
Yes	1.67 (1.20, 2.32)		1.44 (0.91, 2.29)		1.89 (1.13, 3.18)	
No	1.73 (1.49, 2.01)		1.94 (1.56, 2.43)		1.58 (1.28, 1.96)	
Smoking status		0.5799		0.3057		0.1763
Non	1.81 (1.52, 2.15)		2.11 (1.55, 2.87)		1.70 (1.38, 2.09)	
Former	1.51 (1.08, 2.10)		1.49 (1.05, 2.11)		1.82 (0.74, 4.49)	
Current	1.62 (1.18, 2.21)		1.91 (1.37, 2.68)		0.87 (0.45, 1.68)	

Adjusted for sex, age, height, BMI, smoking status, exercise habits, ALT, AST, GGT, HDL-C, TC, TG, FPG, HbA1c and DBP

Note: In each case, the model is not adjusted for the stratification variable

*Abbreviations*: *CI* confidence interval, *OR* Odds ratios; other abbreviations as in Table ​1

### Nonlinear association between WWI and NAFLD

**Fig. 2:**
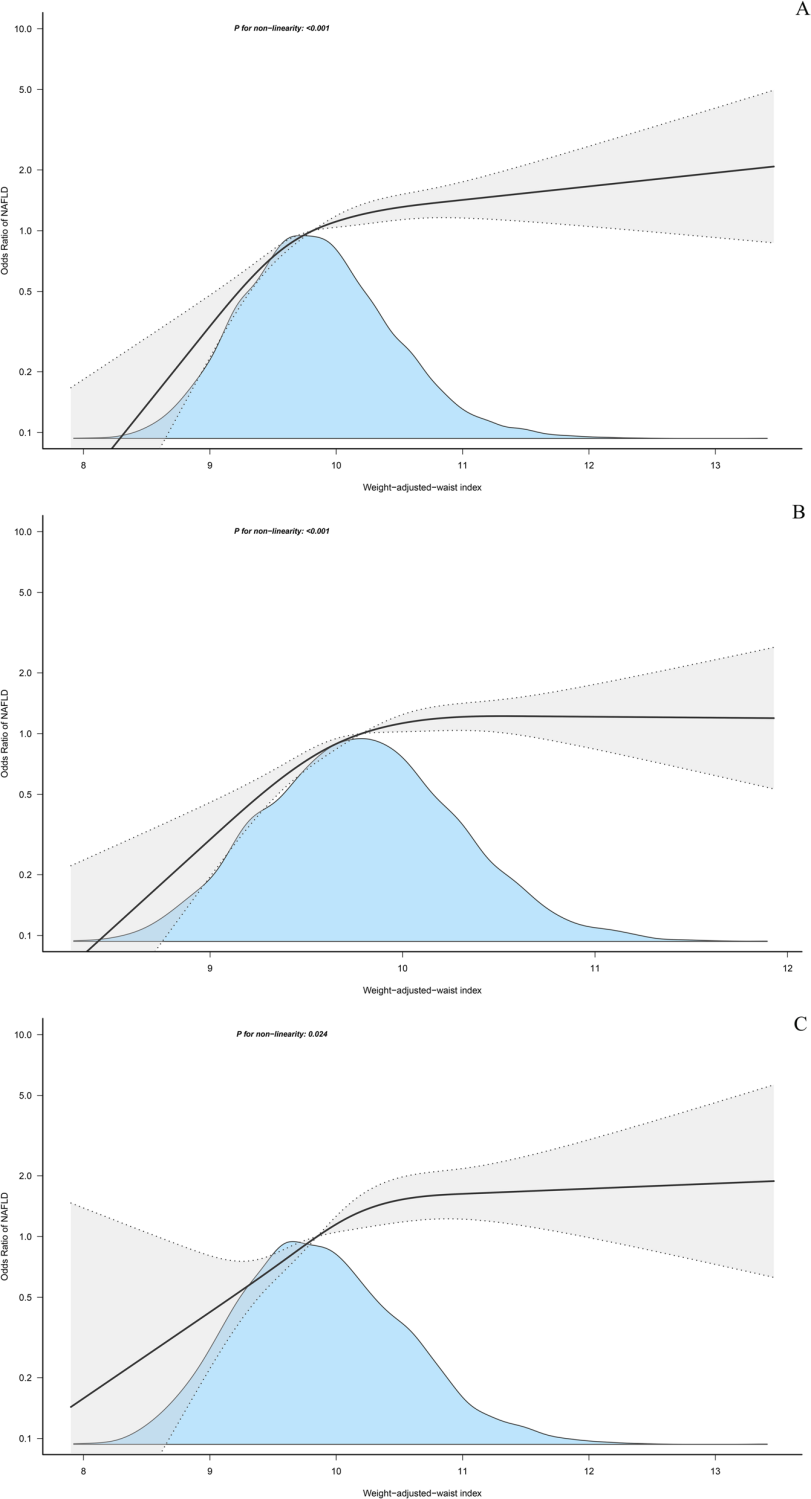
Restricted cubic spline analysis of WWI for the estimation of the risk of NAFLD; **A** all population; **B** women; **C** men. Restricted cubic spline model adjusted for sex, age, height, BMI, drinking status, smoking status, exercise habits, ALT, AST, GGT, HDL-C, TC, TG, FPG, HbA1c and DBP. Note: Sex itself is not adjusted in the analysis based on gender stratification. NAFLD: non-alcoholic fatty liver disease; WWI: weight-adjusted waist index

Using RCS, we further modeled the dose–response relationship of WWI with NAFLD in the whole population and in both sexes. As shown in Fig. 2, after adjusting for all non-collinear variables, we observed that the positive trend between WWI and NAFLD risk remained unchanged. Individuals with higher levels of WWI had a stronger correlation with NAFLD compared to those with lower WWI levels. Furthermore, the RCS analysis indicated a non-linear relationship of NAFLD with WWI (All *P* for non-linearity: < 0.05). It can be observed that in the analysis of the whole population and for both sexes, the increasing trend of the correlation between WWI and NAFLD gradually slowed down when WWI was around 10.

### ROC analysis

Table 5 presents the ROC analysis results of WWI for the identification of NAFLD. The findings revealed that the AUCs of WWI for detecting NAFLD in the entire population, males, and females were 0.6868, 0.7184, and 0.7631, respectively, with corresponding cut-off values of 9.7300, 9.7675, and 9.9987. Compared to the other groups, WWI demonstrated higher accuracy in identifying NAFLD in females, and also had a higher cut-off value.

**Table 5:** AUC, cutoff point, sensitivity and specificity of WWI for identifying NAFLD in both sexes

	**AUC**	**95%CI low**	**95%CI upp**	**Cut-off point**	**Specificity**	**Sensitivity**
**All population**	0.6868	0.6756	0.6981	9.7300	0.4954	0.7974
**Women**	0.7631	0.7421	0.7841	9.9987	0.6166	0.7957
**Men**	0.7184	0.7042	0.7325	9.7675	0.5821	0.7369

*Abbreviations*: *AUC* area under the curve, *NAFLD* non-alcoholic fatty liver disease, *WWI* weight-adjusted-waist index

## Discussion

This observational study involving 11,805 subjects revealed a positive correlation of WWI with NAFLD. This association was consistent across different age groups, exercise habits and smoking status, except for different BMI groups. Notably, the relationship of WWI with NAFLD was stronger in the non-obese population compared to the overweight/obese population.

As far as we know, this is the first study investigating the relationship of WWI with NAFLD. Previous studies have shown that obesity as defined by the traditional obesity index BMI is closely related to the occurrence of NAFLD, and there is a significant dose-dependent relationship [13]. In recent years, with the in-depth study of obesity and NAFLD, people pay more attention to the role of central obesity in the occurrence and development of NAFLD, and people's concept has gradually changed to that central obesity is the key factor leading to the development of NAFLD [1, 12, 14, 35]. In the current study, we revealed an independent correlation between WWI, an index assessing central obesity, and NAFLD. For each unit increase in WWI, the risk of NAFLD increased by 72% in the entire population, by 84% in men, and by 63% in women.

WWI has attracted considerable attention as a novel obesity index since its introduction in 2018. Initially proposed by Park et al., WWI was developed to address the "obesity paradox" observed in cardiovascular disease associated with BMI, while accounting for the high correlation between WC and BMI. They standardized WC by body weight and demonstrated a linear correlation between WWI and cardiovascular-related diseases and mortality rates [21]. Subsequent studies have shown the efficacy of WWI in risk assessment for cardiovascular diseases [24–26] and its potential value in metabolic-related diseases, kidney diseases, and other areas [27–29]. Recently, Shen et al. investigated the association of WWI with liver fat deposition in Americans, further expanding the applications of WWI. They quantified liver fat deposition and fibrosis using vibration-controlled transient elastography and found a significant positive correlation of WWI with liver fat deposition. Moreover, by fitting the smooth curve, they also found a U-shaped nonlinear correlation of WWI with liver fibrosis, and on both sides of WWI equal to 10.92, the WWI-related liver fibrosis risk showed an opposite trend [30]. Building upon Shen et al.'s study, our research further evaluated the association of WWI with NAFLD, yielding significant positive associations. These findings provided further support for Shen et al.'s results [30], suggesting that WWI was a useful index for evaluating liver fat deposition. However, it is noteworthy that our study also identified a nonlinear relationship between WWI and NAFLD; whether in the entire population, men or women, there was a gradual attenuation of the positive association when WWI approached 10, which contrasted with Shen et al.'s findings.

The underlying mechanisms linking WWI and NAFLD remain unclear. Several possible explanations included: (i) WWI reflects central obesity, which actively contributes to adipocyte dysfunction, insulin resistance, and chronic inflammation, all of which further promote the development of NAFLD [10, 36]. (ii) WWI is positively correlated with the visceral fat area, and visceral fat, due to its unique anatomical location, releases metabolites that more easily reach the liver, thus affecting hepatic fat metabolism [37]. (iii) WWI reflects muscle mass reduction. Similar to the mechanisms of obesity, infiltration of adipose tissue into skeletal muscle is associated with insulin resistance and chronic inflammation, both of which contribute to NAFLD development [38, 39].

It is important to note that, in the current analysis subgroup of the research, whether in the whole population or in the men and women, the correlation between WWI and NAFLD was found to be particularly stronger in non-obese individuals. In fact, "lean-type" NAFLD has garnered increasing attention from scholars in recent years [40]. According to the report, about 40% of people with NAFLD worldwide are classified as non-obese, and nearly one in five of them are thin [41]. Researchers have conducted several studies in non-obese populations to explore the potential mechanisms underlying the susceptibility to NAFLD. These mechanisms may include: (i) genetic predisposition is considered the primary cause of "lean-type" NAFLD. The PNPLA3 gene has been identified as a genetic determinant of NAFLD, and lean individuals are more susceptible to the effects of PNPLA3 gene polymorphisms, increasing their likelihood of carrying risk alleles compared to overweight and obese individuals [42–44]. (ii) Diet is an important factor in NAFLD. Studies have found that lean individuals tend to consume more fructose and cholesterol, which will promote the occurrence of NAFLD [43, 44]. (iii) Other factors such as metabolic syndrome, disrupted gut microbiota, and decreased skeletal muscle mass and function have also been implicated in the formation of non-obese NAFLD [42–44]. It is noteworthy that non-obese NAFLD patients are not at low risk for overall mortality, cardiovascular-related mortality, or liver-related mortality [41, 45]. Therefore, while focusing on the obese population, attention should also be paid to the occurrence of NAFLD in non-obese individuals.

### Strengths and limitations

#### Strengths

(i) For the first time, we found a correlation between a new obesity indicator, WWI, and NAFLD, and these findings can provide new insights into the risk management of NAFLD. (ii) WWI is easy to calculate and suitable for practical applications. Moreover, the NAGALA project's samples were drawn from a general health check-up population, and it had a sufficient sample size, making it applicable for promotion among ordinary people. (iii) This study employed rigorous statistical methods to adjust for non-collinear variables and performed four sensitivity analyses, indicating the reliability of the research findings.

#### Limitations

(i) The NAGALA study was conducted in a Japanese population, and further investigation is needed to determine its applicability in other regions or populations. (ii) This study was cross-sectional in nature and cannot establish a causal association of WWI with NAFLD. (iii) The diagnosis of NAFLD in this study was based on abdominal ultrasonography, which may underestimate liver fat content in some patients [46]. (iv) Although we included and controlled for known covariates, there still may be unmeasured confounding variables that could interfere with the conclusions of this study.

## Conclusions

The current study demonstrated a positive and nonlinear association of WWI with NAFLD, with a more pronounced effect of WWI on NAFLD risk in non-obese individuals compared to overweight or obese individuals. These findings provided valuable new reference information for the risk assessment of NAFLD and added new evidence to its prevention.

### Supplementary Information

**Additional file 1: Supplementary Table 1.** Collinearity diagnostics steps.

## Data Availability

The data used in this study have been uploaded to the "Dryad" database by Professor Okamura et al. (10.5061/dryad.8q0p192).

## Abbreviations

NAFLD: Non-alcoholic fatty liver disease
WWI: Weight-adjusted waist index
ROC: Receiver operating characteristic
TC: Total cholesterol
HDL-C: High-density lipoprotein cholesterol
LDL-C: Low-density lipoprotein cholesterol
TG: Triglyceride
WC: Waist circumference
BMI: Body mass index
GGT: γ-Glutamyl transferase
AST: Aspartate aminotransferase
HbA1c: Glycosylated hemoglobin A1c
FPG: Fasting glucose
ALT: Alanine aminotransferase
OR: Odds ratio
CI: Confidence intervals
RCS: Restricted cubic spline
