# Supplementary material for: Association between weight-adjusted waist index and non-alcoholic fatty liver disease: a population-based study
Source: BMC Endocr Disord. 2024 Feb 18;24:22. doi: 10.1186/s12902-024-01554-z (PMC10874525; doi:10.1186/s12902-024-01554-z)
Supplement: Supplementary file 1 — Additional file 1: Supplementary Table 1. Collinearity diagnostics steps. [file 12902_2024_1554_MOESM1_ESM.docx]

Supplementary Table 1: Collinearity diagnostics steps.

|  | Variance inflation factor | | | |
| --- | --- | --- | --- | --- |
|  | Step 1 | Step 2 | Step 3 | Step 4 |
| WWI | 134.3 | 1.5 | 1.4 | 1.4 |
| Sex | 3.1 | 3.1 | 3.1 | 3.1 |
| Age | 1.3 | 1.3 | 1.3 | 1.3 |
| Weight | 212.7 | 167.3 | NA | NA |
| Height | 88.3 | 49.7 | 2.5 | 2.5 |
| BMI | 173.3 | 93.3 | 1.6 | 1.6 |
| WC | 544.1 | NA | NA | NA |
| ALT | 4.4 | 4.3 | 4.3 | 4.3 |
| AST | 3.5 | 3.5 | 3.5 | 3.5 |
| GGT | 1.4 | 1.4 | 1.4 | 1.4 |
| HDL-C | 1.8 | 1.8 | 1.8 | 1.8 |
| TC | 1.5 | 1.5 | 1.5 | 1.5 |
| TG | 1.7 | 1.7 | 1.7 | 1.7 |
| HbA1c | 1.1 | 1.1 | 1.1 | 1.1 |
| FPG | 1.1 | 1.1 | 1.1 | 1.1 |
| SBP | 5.5 | 5.5 | 5.5 | NA |
| DBP | 5.4 | 5.4 | 5.4 | 1.1 |
| Exercise habits | 1 | 1 | 1 | 1 |
| Smoking status | 1.3 | 1.3 | 1.3 | 1.3 |

Note-1: Variance inflation factor = 1/(1-R^2^). Abbreviations as in Table 1.

Note-2: The variables with Variance inflation factor >5 will be regarded as collinear variables and cannot be included in the multiple regression model.
